# Negative pressure pulmonary edema in a 2-month-old infant after general anesthesia: a case report

**DOI:** 10.1186/s40981-023-00670-4

**Published:** 2023-11-15

**Authors:** Yusuke Miyazaki, Yoshie Taniguchi, Chika Miyazaki, Elissa Allen, Fumina Yoshimoto

**Affiliations:** Department of Anesthesiology, The Jikei University School of Medicine, Tokyo, Japan

To the Editor,

We report a rare case of negative pressure pulmonary edema (NPPE) following extubation in a 2-month-old male infant (height 60.5 cm, weight 5.0 kg). Born full-term and healthy, he developed vomiting a week prior to admission. Diagnosed with hypertrophic pyloric stenosis, he underwent pyloromyotomy on his 2-month birthday. On entering the operating room, stomach contents were aspirated using an indwelling gastric tube. Following rapid sequence induction with propofol 20 mg, fentanyl 15 μg, and rocuronium 5 mg, a smooth and successful first-attempt intubation was achieved using a 3.5 mm uncuffed tube and a video laryngoscope (Airway Scope; Pentax), obtaining a Cormack-Lehane grade of 1. No laryngeal anatomical abnormalities were noted. An air leak at 20 cmH_2_O was confirmed. Anesthesia was maintained with sevoflurane, fentanyl, and rocuronium. After confirming adequate recovery from neuromuscular blockade via sugammadex through quantitative neuromuscular monitoring and ensuring adequate spontaneous ventilation, the patient was extubated post-surgery. After extubation, the patient exhibited increased inspiratory effort accompanied by inspiratory stridor and paradoxical chest and abdominal movements. Shortly afterward, SpO_2_ dropped to below 70% and heart rate decreased to 75 beats/min. Despite manual ventilation using an oral airway with 100% O_2_ and continuous positive airway pressure (PAP), SpO_2_ did not improve, leading to suspicion of laryngospasm. After propofol and atropine was administered, SpO_2_ increased to above 98%, but coarse crackles were detected in both lungs, and a pink frothy fluid was noted in the mouth. A subsequent X-ray showed bilateral diffuse pulmonary infiltration (Fig. 1A), leading to a diagnosis of NPPE. The patient was re-intubated with a 3.5 mm uncuffed tube. Laryngeal swelling and erythema were observed, and blood-tinged frothy sputum was suctioned from the trachea. A post-reintubation X-ray showed decreased diffuse pulmonary infiltration (Fig. 1B). Subsequently, the patient was transferred to the pediatric intensive care unit (PICU) and ventilated with bilevel PAP (FiO_2_ 0.3; inspiratory/expiratory PAP 16/4 cmH_2_O). The blood gas analysis showed the following: pH 7.357, PaO_2_ 109 mmHg, PaCO_2_ 42.5 mmHg, and HCO_3_^−^ 23.8 mEq/L. Dexamethasone 0.15 mg/kg was administered thrice at 6-h intervals, leading to extubation 17 h post-PICU admission. After extubation, he received high-flow nasal cannula (FiO_2_ 0.25; 10 L/min) for 12 h before ceasing oxygen administration. He moved to the general ward on postoperative day 2 and was discharged uneventfully on day 4.

**Fig. 1:**
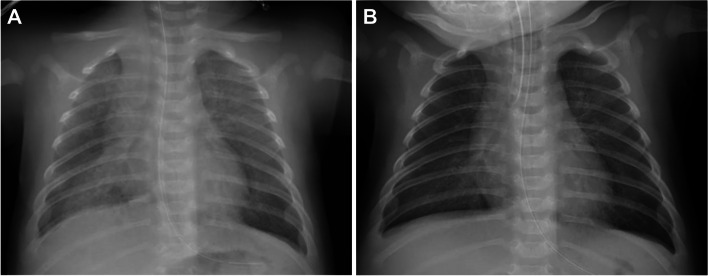
**A** Chest radiograph obtained in the operating room 15 min after extubation, showing bilateral diffuse pulmonary infiltration. **B** Chest radiograph 9 min after re-intubation, showing an improvement in diffuse pulmonary infiltration

To our knowledge, this is the first reported case of NPPE in a 2-month-old infant with no pre-existing respiratory tract diseases, occurring due to upper airway obstruction following extubation. The case illustrates that postextubation NPPE can occur even in infants, not just in older children and adults capable of generating larger negative intrathoracic pressure. While NPPE typically resolves within 12–48 h with appropriate early intervention, 11–40% of NPPE cases can become severe [1–3]. Therefore, the prevention of NPPE is crucial. Further research is needed to investigate the extent to which factors potentially affecting post-extubation stridor or laryngospasm—such as the choice between cuffed and uncuffed endotracheal tubes, or the use of sugammadex—impact the incidence of NPPE [4–7].

## Data Availability

The data used in this report are available from the corresponding author upon reasonable request.

## Abbreviations

FiO_2_: Fraction of inspired oxygen concentration
NPPE: Negative pressure pulmonary edema
PAP: Positive airway pressure
PICU: Pediatric intensive care unit
SpO_2_: Oxygen saturation measured by pulse oximetry
